# Cisplatin Targeting of Bacterial Ribosomal RNA Hairpins

**DOI:** 10.3390/ijms160921392

**Published:** 2015-09-07

**Authors:** Gayani N. P. Dedduwa-Mudalige, Christine S. Chow

**Affiliations:** Department of Chemistry, Wayne State University, Detroit, MI 48202, USA; E-Mail: dedduwa.mudalige@wayne.edu

**Keywords:** cisplatin, ribosomal RNA, helix 69, helix 24

## Abstract

Cisplatin is a clinically important chemotherapeutic agent known to target purine bases in nucleic acids. In addition to major deoxyribonucleic acid (DNA) intrastrand cross-links, cisplatin also forms stable adducts with many types of ribonucleic acid (RNA) including siRNA, spliceosomal RNAs, tRNA, and rRNA. All of these RNAs play vital roles in the cell, such as catalysis of protein synthesis by rRNA, and therefore serve as potential drug targets. This work focused on platination of two highly conserved RNA hairpins from *E. coli* ribosomes, namely pseudouridine-modified helix 69 from 23S rRNA and the 790 loop of helix 24 from 16S rRNA. RNase T1 probing, MALDI mass spectrometry, and dimethyl sulfate mapping revealed platination at GpG sites. Chemical probing results also showed platination-induced RNA structural changes. These findings reveal solvent and structural accessibility of sites within bacterial RNA secondary structures that are functionally significant and therefore viable targets for cisplatin as well as other classes of small molecules. Identifying target preferences at the nucleotide level, as well as determining cisplatin-induced RNA conformational changes, is important for the design of more potent drug molecules. Furthermore, the knowledge gained through studies of RNA-targeting by cisplatin is applicable to a broad range of organisms from bacteria to human.

## 1. Introduction

**Figure 1:**
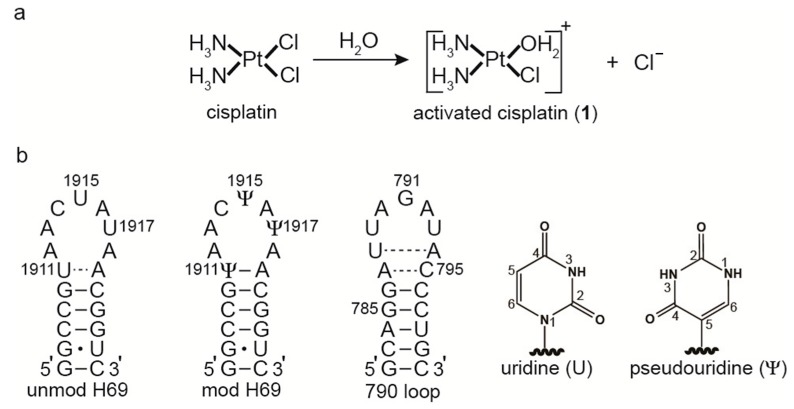
Cisplatin and model rRNA constructs. (**a**) *Cis*-diamminedichloridoplatinum(II) forms *cis*-diammine(aqua)chloridoplatinum(II), **1**, in water by displacement of a chlorido ligand; (**b**) Three RNA constructs were used: unmodified H69 with uridines at 1911, 1915, and 1917 (*E. coli* numbering); modified H69 containing pseudouridine (Ψ) at the same positions; and the 790 loop. The 790 loop contains an additional G-C base pair (782–800). The base structure of Ψ is compared to uridine.

Since its discovery as an anticancer agent in the 1960s, *cis*-diamminedichloridoplatinum(II), or cisplatin, has been used to treat a variety of human carcinomas [1,2]. The cytotoxic activity of this platinum-based compound is believed to be associated with deoxyribonucleic acid (DNA) lesions that influence key cellular functions [2]. Cisplatin is a neutral molecule that becomes positively charged upon displacement of a chlorido ligand with water to form the active monoaquated complex **1** (Figure 1) [3,4]. The positive charge on **1** directs it to negatively charged nucleic acids. The active species coordinates to the N7 of purine bases and forms stable adducts on DNA [5,6]. Although DNA is considered to be the major target of cisplatin, ribonucleic acid (RNA) and proteins are also susceptible to platinum-adduct formation [7,8,9,10,11]. Recent studies directed towards understanding RNA-cisplatin interactions have revealed that different cellular RNAs are targets of cisplatin and its analogs [12,13,14,15,16,17,18].

Previous studies to determine the Pt(II) distribution in cells indicated rRNA as one of the major targets of cisplatin [16]. Cisplatin and several of its analogs also impact ribosome-related activities such as subunit association and translation [8,9,11]. In addition, cisplatin cytotoxicity was shown to be modulated by compounds that target the ribosome and inhibit protein synthesis [19]. Therefore, even if the ribosome itself does not have a direct role in the anticancer activity of cisplatin, it may mediate drug toxicity and resistance. In contrast to work with DNA, the RNA-Pt(II) adducts have not been well characterized. Due to differences in their secondary structures, such as groove widths, sugar puckers, and backbone phosphate-phosphate distances, DNA and RNA may have different adduct profiles and/or nucleotide preferences. Although rRNA also has a complex tertiary structure, X-ray crystallographic and NMR studies have shown that several of its secondary structure motifs are in fact good representations of the full-length rRNA structures within the contexts of individual subunits, including dynamic structural changes that occur upon drug binding or subunit association [20,21]. The goal of this study was to examine several functionally important motifs that reside in the subunit interface and are known to play key roles in protein synthesis as well as drug interactions. The chosen motifs also have highly conserved sequences that include GpG sites, which are predicted to be targets of cisplatin. In this case, bacterial models were employed due to the availability of high-resolution X-ray structures as well as solution structures [22,23,24]; however, we believe that the results regarding adduct preferences can be applied to other organisms because of high rRNA sequence conservation across phylogeny.

In this work, reactions of complex **1** with three rRNA constructs representing helix 69 (H69) and the 790 loop of *E. coli* ribosomes were carried out (Figure 1). Ribosomes have three important functional regions, namely the decoding region, the peptidyltransferase center, and the subunit interface, which all involve unique RNA structural motifs [22,25]. At the subunit interface, helix 69 in the large subunit and the 790 loop of helix 24 (h24) in the small subunit both play key roles in translation. Helix 69 is located in domain IV of bacterial 23S rRNA and has a highly conserved nucleotide sequence [26]. Together with helix 44 of the small subunit, H69 forms bridge B2a of complete 70S ribosomes [25]. Moreover, H69 is involved in translation initiation and ribosome recycling [27,28,29]. Studies on small RNA constructs, as well as full-length 23S rRNA in 50S and 70S ribosomes, showed that H69 undergoes conformational changes that are dependent upon the natural pseudouridine (Ψ, Figure 1) modifications at positions 1911, 1915, and 1917 (*E. coli* numbering) [30,31,32]. The 790 loop is located in the central domain of 16S rRNA and also contains a highly conserved nucleotide sequence [33]. Previous studies revealed that this region is exposed on the surface of the 30S subunit and directly involved in subunit association and translation initiation [24,34,35,36]. Both of these RNA motifs are known target sites for small molecules [37,38].

A combination of chemical and enzymatic probing can be used to interrogate modified or metalated RNAs. RNase T1 causes hydrolysis of the RNA backbone at G residues [39]. Dimethyl sulfate (DMS) reacts primarily with G N7, A N1, and to a lesser extent C N3 sites [40]. Further chemical treatment with sodium borohydride and aniline leads to strand scission at G sites that are modified at N7. Either matrix-assisted laser desorption-ionization time-of-flight mass spectrometry (MALDI MS) or ^32^P-end labeling combined with denaturing polyacrylamide gel electrophoresis can be used to identify the number of platination events as well as the sites of adduct formation on RNA fragments.

The influence from pseudouridine (Ψ) modifications on platination of rRNA was also investigated by using the model rRNA hairpins. Platination of unmodified H69, which contains uridines at positions 1911, 1915, and 1917 (*E. coli* numbering), was compared to that of modified H69 possessing Ψs at the same positions. The impact of nucleotide sequence on cisplatin coordination was evaluated by using the 790 loop, which contains an identical type and number of nucleotides (A_5_G_5_U_4_C_5_) as unmodified H69. Our results revealed that complex **1** forms two adducts with H69 RNAs and one adduct with the 790 loop. Mass spectrometry, RNase T1 digestion, and chemical-probing analysis suggested consecutive Gs as the most plausible platination sites in all three rRNA hairpins. The DMS-probing experiments also revealed structural changes induced by cisplatin coordination to the H69 rRNA hairpins. This work suggests that H69 rRNA and the 790 loop are potential cisplatin targets in *E. coli* ribosomes, and adduct formation could possibly interfere with ribosome function and contribute to drug cytotoxicity in bacteria as well as other organisms due to high sequence conservation.

## 2. Results and Discussion

### 2.1. Matrix-Assisted Laser Desorption-Ionization Time-of-Flight (MALDI) Mass Spectrometry and RNase T1 Mapping Studies

A number of studies revealed RNA as a target for platinum-based drugs, as well as other drug classes. In this study, the coordination of cisplatin with three 19-nucleotide model rRNAs hairpins representing *E. coli* H69 (unmodified and modified) and the 790 loop was investigated. Comparisons were made with unmodified H69 to determine whether Ψs (modified H69) or altered nucleotide sequences (790 loop) would influence platination. The data indicate minimal influence of Ψs and sequence for platination target selectivity; however, structural and/or stability effects on residues distant to the target sites were observed, and a possible sequence influence to this effect was identified.

**Figure 2:**
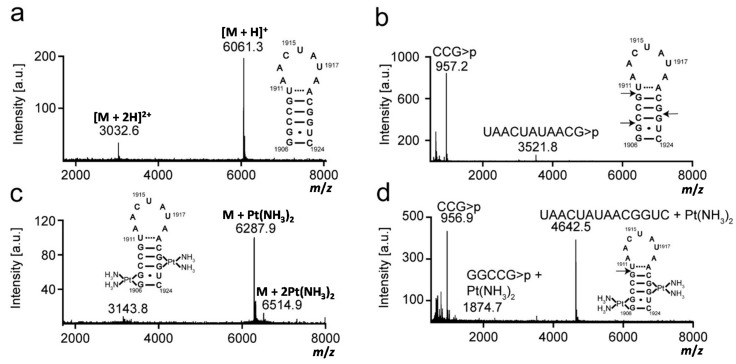
Mass analysis of platinated unmodified H69 is shown. Mass spectra of (**a**) unmodified H69 parent RNA (unplatinated H69); (**b**) RNase T1 digestion of unplatinated H69; (**c**) platinated unmodified H69; and (**d**) RNase T1 digestion of platinated unmodified H69 are given. Arrows indicate RNase T1 cleavage sites.

**Figure 3:**
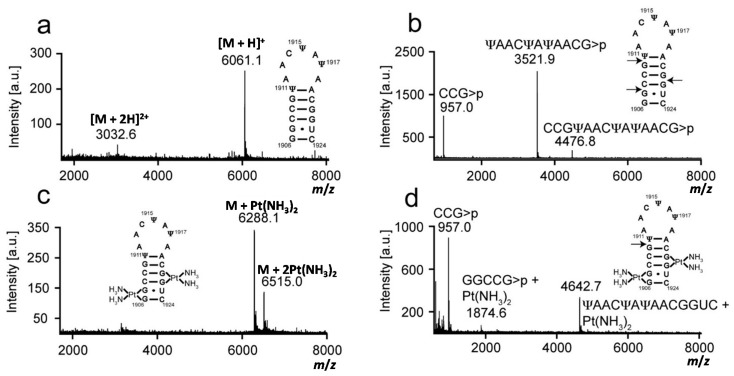
Mass analysis of platinated modified H69 is shown. Mass spectra of (**a**) modified H69 parent RNA (unplatinated RNA); (**b**) RNase T1 digestion of unplatinated H69; (**c**) platinated modified H69; and (**d**) RNase T1 digestion of platinated modified H69 are given. Arrows indicate RNase T1 cleavage sites.

**Figure 4:**
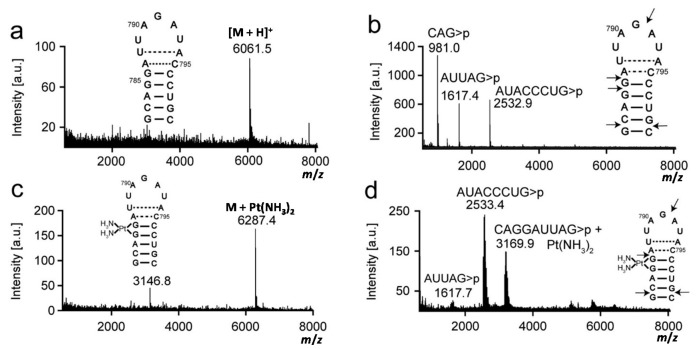
Mass analysis of platinated 790 loop is shown. Mass spectra of (**a**) 790 loop parent strand (unplatinated 790 loop); (**b**) RNase T1 digestion of unplatinated 790 loop; (**c**) platinated 790 loop; and (**d**) RNase T1 digestion of platinated 790 loop are shown. Arrows indicate RNase T1 cleavage sites.

Platination was carried out between complex **1** and rRNA hairpins, and the products were isolated by gel electrophoresis and characterized by MALDI MS to first determine the number of adducts. Mass data were collected in the positive-ion mode, and analysis of the H69 and 790 RNAs produced [M + H]^+^ and [M + 2H]^2+^ peaks. The experimental and expected masses of full-length RNA molecules are listed in Table 1 and shown in Figure 2, Figure 3 and Figure 4. Following the reaction with complex **1**, two molecular ion peaks ([M + Pt-H]^+^ or [M + 2Pt-3H]^+^) were observed for both unmodified and modified H69 (Figure 2 and Figure 3, respectively). Platination of the 790 loop produced a single molecular ion peak ([M + Pt-H]^+^) (Figure 4). The experimental masses obtained correspond well to the predicted masses. An increase in mass by 229 or 458 Da corresponds to coordination of one or two Pt(NH_3_)_2_ moieties to the RNA, respectively, with loss of the aqua and chlorido ligands, indicating one or two platination sites for each RNA [41,42]. This observation is supported by the addition of a Pt(NH_3_)_2_ group to DNA detected by MALDI MS [43,44].

Next, platination sites were determined through RNase T1 digestion. Mass increases for the fragments obtained from enzyme digestion of platinated RNAs indicated the general locations of the adducts. The experimental and expected masses of the RNase T1 fragments are listed in Table 1 and shown in Figure 2, Figure 3 and Figure 4. RNase T1 digestion occurs via a 2′,3′–cyclic phosphate intermediate [39]. Therefore, under partial digestion conditions, RNase T1 treatment led to RNA fragments containing either a 2′, 3′, or 2′,3′–cyclic phosphate (indicated as >p). The digestion of H69 RNA produced mass peaks corresponding to fragments CCG>p, (U/Ψ)AAC(U/Ψ)A(U/Ψ)AACG>p, and CCGΨAACΨAΨAACG>p (in modified H69 only). RNase T1 digestion of the 790 loop generated mass peaks assigned as fragments CAG>p, AUUAG>p, and AUACCCUG>p. In contrast, the mass spectrum obtained after treatment of platinated H69 RNAs revealed two new peaks corresponding to GGCCG>p + Pt(NH_3_)_2_ and (U/Ψ)AAC(U/Ψ)A(U/Ψ)AACGGUC + Pt(NH_3_)_2_. RNase T1 digestion of the platinated 790 loop produced a new peak corresponding to CAGGAUUAG>p + Pt(NH_3_)_2_. The production of RNase T1 fragments CCG>p, (U/Ψ)AAC(U/Ψ)A(U/Ψ)AACG>p, and CCGΨAACΨAΨAACG>p (modified H69 only) of H69 RNAs indicated G1907, G1910 and G1921 as the enzyme cleavage sites (Figure 2 and Figure 3). In contrast, the absence of fragments resulting from cleavage between G1906-07 or G1921-22, as well as production of GGCCG>p and (U/Ψ)AAC(U/Ψ)A(U/Ψ)AACGGUC fragments with a 229 Da mass increase, indicated G1906-07 and G1921-22 as the most plausible platinum target sites on H69 RNAs. The production of GGCCG>p with Pt(NH_3_)_2_ revealed that G1910 was still available for RNase T1 cleavage and not a likely platination site. Combined, these results demonstrated that the presence of Ψ did not alter the site preference of **1** for H69 RNAs, and GpGs were the most likely platination sites in these rRNA hairpins.

The production of CAG>p, AUUAG>p and AUACCCUG>p fragments by RNase T1 digestion on the 790 loop indicated G782, G785, G786, G791, and G799 as the enzyme cleavage sites (Figure 4). Following platination and digestion of the 790 loop, the AUACCCUG>p fragment was observed, indicating cleavage at G791 and G799. In contrast, the intensity of the AUUAG>p fragment peak diminished, suggesting blockage of RNase T1 cleavage at G786. Production of the CAGGAUUAG>p fragment with an increased mass of 229 Da, and blocked RNase T1 cleavage with concomitant loss of the CAG>p fragment suggested platination at G785-86. The reaction at G782 was not blocked, thus platination at this site is not likely. All of the RNase T1 fragments resulting from platinated RNA digestion contain consecutive G residues (G1906-G1907 and G1921-G1922 on H69, and G785-G786 on the 790 loop), revealing that GpG sites are preferred for complex **1** coordination to the studied rRNA constructs. However, we could not rule out GpA as a potential reactive site by this approach.

**Table 1:** Predicted and experimental masses of unmodified H69, modified H69, and 790 loop RNA (parent strands, platinated products, and RNase T1 digestion fragments).

Construct	[RNA]^+^	Predicted Mass ^a^ *m*/*z*	Experimental Mass *m*/*z*
unmodified H69	Parent strand + H^+^	6061.7	6061.3
	Parent strand + [Pt(NH_3_)_2_]^2+^ − H^+^	6288.7	6287.9
	Parent strand + 2[Pt(NH_3_)_2_]^2+^ − 3H^+^	6515.7	6514.9
	5′CCG>p3′ + H^+^	956.6	957.2/956.9 ^b^
	5′UAACUAUAACG>p3′ + H^+^	3521.1	3521.8
	5′GGCCG>p3′ + [Pt(NH_3_)_2_]^2+^ − H^+^	1873.9	1874.7
	5′UAACUAUAACGGUC3′ + [Pt(NH_3_)_2_]^2+^ − H^+^	4642.7	4642.5
modified H69	Parent strand + H^+^	6061.7	6061.1
	Parent strand + [Pt(NH_3_)_2_]^2+^ − H^+^	6288.7	6288.1
	Parent strand + 2[Pt(NH_3_)_2_]^2+^ − 3H^+^	6515.7	6515.0
	5′CCG>p3′ + H^+^	956.6	957.0/957.0 ^b^
	5′ΨAACΨAΨAACG>p3′ + H^+^	3521.1	3521.9
	5′CCGΨAACΨAΨAACG>p3′ + H^+^	4476.7	4476.8
	5′GGCCG>p3′ + [Pt(NH_3_)_2_]^2+^ − H^+^	1874.0	1874.6
	5′ΨAACΨAΨAACGGUC3′ + [Pt(NH_3_)_2_]^2+^ − H^+^	4642.7	4642.7
790 loop	Parent strand + H^+^ Parent strand + [Pt(NH_3_)_2_]^2+^ − H^+^	6061.7 6288.7	6061.5 6287.4
	5′CAG>p3′ + H^+^	980.6	981.0
	5′AUUAG>p3′ + H^+^	1616.9	1617.4/1617.7 ^b^
	5′AUACCCUG>p3′ + H^+^	2532.5	2532.9/2533.4 ^b^
	5′CAGGAUUAG>p3′ + [Pt(NH_3_)_2_]^2+^ − H^+^	3168.7	3169.9

^a^ The expected mass values for the parent RNA constructs and the digestion fragments were obtained from Mongo Oligo Calculator v2.06 (http://mods.rna.albany.edu/masspec/Mongo-Oligo); ^b^ *m*/*z* values correspond to fragments derived from unplatinated/platinated RNA. The production of cyclic phosphates at the 3′ ends (or 2′ or 3′ phosphates) from RNase T1 digestion is denoted as >p.

### 2.2. Dimethyl Sulfate Probing of Platination Sites

Guanine-specific chemical reactions were carried out to provide further evidence of platination of the H69 and 790 loop RNAs at nucleotide resolution. The RNAs were treated with DMS to methylate the accessible G N7 positions followed by sodium borohydride and aniline treatments to initiate strand scission and allow identification of the modified sites [45]. In cisplatin reactions with DNA, platination occurs at G N7 [5]. Therefore, similar adduct formation on H69 or the 790 loop was expected to prevent DMS methylation and cause a disappearance of cleavage bands in sequencing gels [12,46].

**Figure 5:**
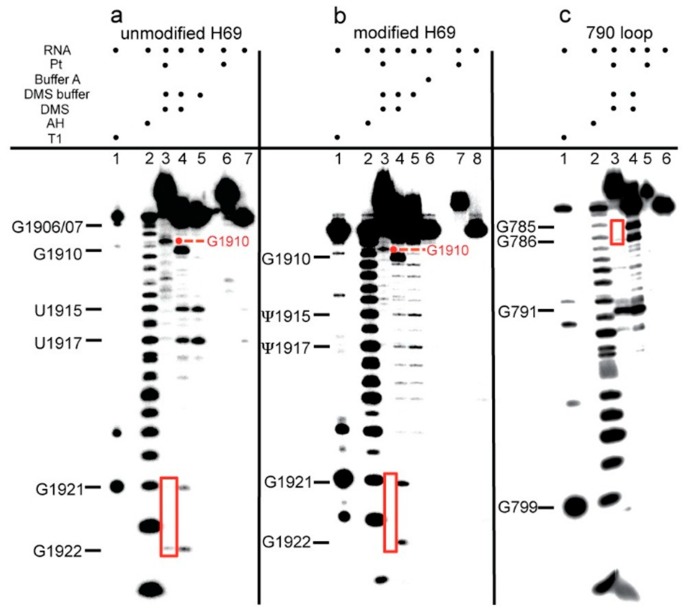
DMS probing of H69 and the 790 loop is shown. Autoradiograms show the results for 3′-end-labeled (**a**) unmodified H69; (**b**) modified H69; and (**c**) 790 loop (T1: RNase T1 reaction on unplatinated RNA; AH: alkaline hydrolysis ladder on unplatinated RNA). Note that T1 and AH fragments migrate slower than the DMS products due to different chemical composition of the ends (5′ OH *vs.* 5′ phosphate, respectively). In panels **a** and **b**, the fragments corresponding to DMS reaction and cleavage at G1910 migrate more slowly in lane 3 compared to lane 4 because of the presence of the platinum adduct. Guanine residues that show slower mobility (dots) or decreased DMS reactivity (boxes) due to platination are indicated in red.

**Figure 6:**
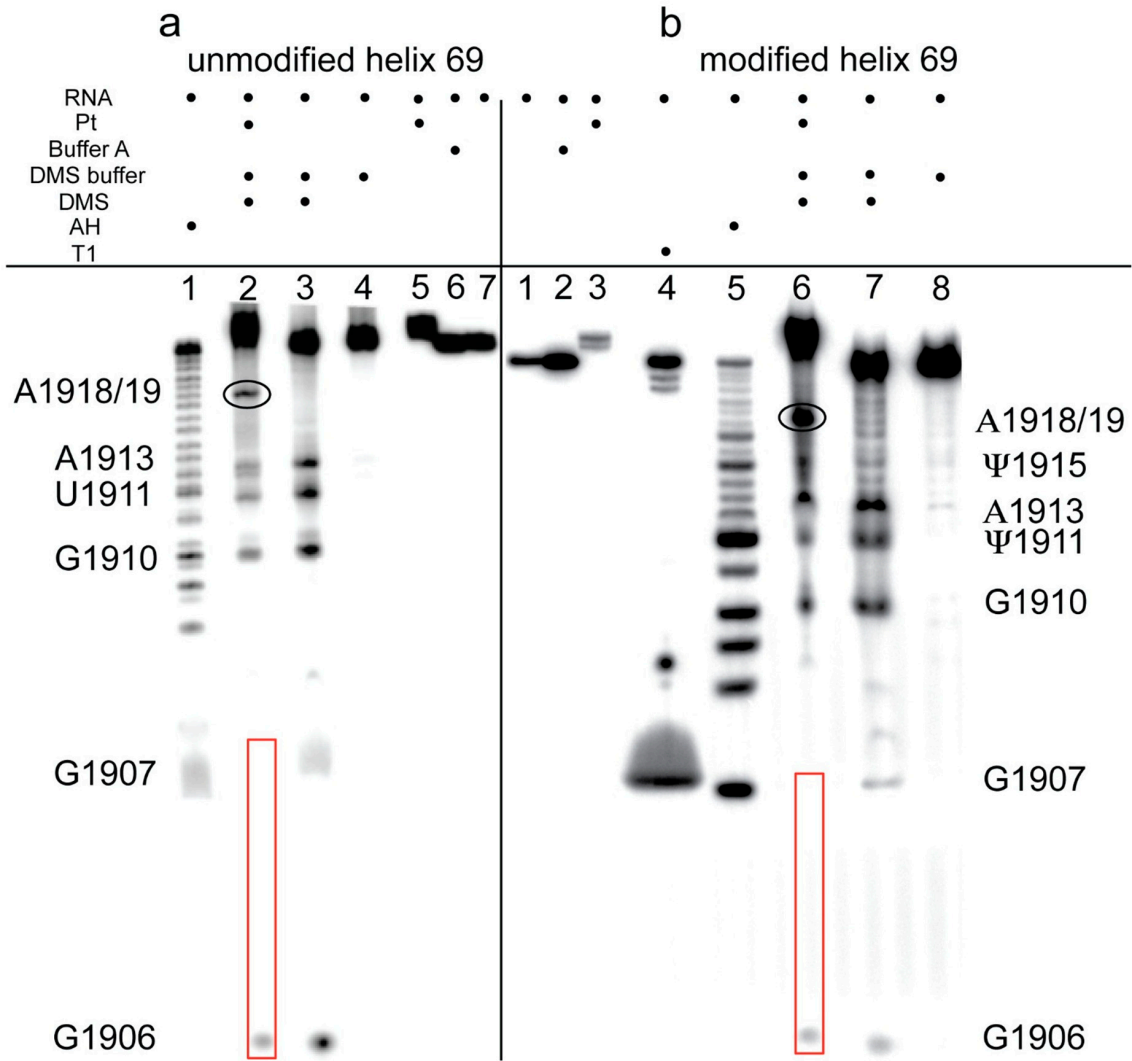
Dimethyl sulfate (DMS) probing of H69 using 5′-labeled RNA is shown. Autoradiograms show the DMS-probing results on 5′-end-labeled (**a**) unmodified H69 and (**b**) modified H69 (T1: RNase T1 reaction on unplatinated RNA; AH: alkaline hydrolysis ladder on unplatinated RNA). Guanine residues that show differences due to platination are indicated with red boxes. Residue A1918/19 (circled) is more sensitive to DMS reactivity following platination.

DMS probing was carried out on unplatinated and platinated versions of H69 and the 790 loop. Autoradiograms of 20% denaturing polyacrylamide gels containing DMS-treated, 3′-end-labeled RNA constructs are shown in Figure 5. Bands corresponding to G1921 and G1922 of H69 and G785 and G786 of the 790 loop were diminished significantly in the DMS-treated platinated RNA samples (Figure 5, panels a–c; see lane 3 compared to lane 4). Due to poor band resolution at the 5′ end of the 3′-labeled RNA, DMS probing was also carried out using 5′-labeled H69 RNAs, which revealed platination at G1906 and G1907 (Figure 6; panel a, lane 2; panel b, lane 6). DMS treatment led to methylation and cleavage at G1910 for both platinated and unplatinated H69 (Figure 5, panels a and b, lanes 3 and 4), indicating that G1910 is not a platination site, although it should be noted that the cleavage products shift (*i.e*., slower mobility) due to presence of the platinum adduct at the 5′ end. Similarly, DMS treatment of the 790 loop produced a cleavage band at G791 (Figure 5, panel c, lanes 3 and 4), indicating the absence of a platinum adduct at this site. In this case, the DMS-cleavage products of both platinum-treated and non-treated samples were observed to co-migrate, since these RNA fragments did not contain any platinum adducts. Together, these results support a model in which complex **1** reacts preferentially at consecutive Gs in RNA (G1906-G1907 and G1921-G1922 for H69 and G785-G786 for the 790 loop) and forms stable bis-adducts (intrastrand adduct type 1,2-GpG), as observed with DNA (1,2-d(GpG)) [47].

In the determination of platinum-coordination sites on 3′-labeled H69 through DMS reactions, unexpected hydrolysis (likely due to contaminating RNases, although highly reproducible) was observed at U/Ψ1915 and U/Ψ1917 (Figure 5, panels a and b, lanes 4 and 5). The hydrolysis mechanism for these two products is supported by the fact that the bands migrate with the alkaline hydrolysis products in lane 2. Hydrolysis at these sites did not occur on the platinated H69 (Figure 5; panel a, lanes 3 and 6; panel b, lanes 3 and 7), suggesting that platination alters the structure of H69 at or near these residues. In contrast, reactivity at U/Ψ1911 and A1913 was observed in DMS probing of 5′-labeled H69 for both platinated and unplatinated RNAs (Figure 6; panel a, lanes 2 and 3; panel b, lanes 6 and 7), indicating a minimal influence of platination on DMS-sensitivities of these nucleotides. However, a new fragment band corresponding to either A1918 or A1919 appeared in the DMS-treated, platinated H69 RNA samples (Figure 6; panel a, lane 2; panel b, lane 6). Collectively, these observations demonstrate that certain H69 residues become either more or less reactive towards DMS, as well as having altered sensitivity to hydrolysis, following platination with complex **1**, indicating different responses of the RNA nucleotides to drug binding.

**Figure 7:**
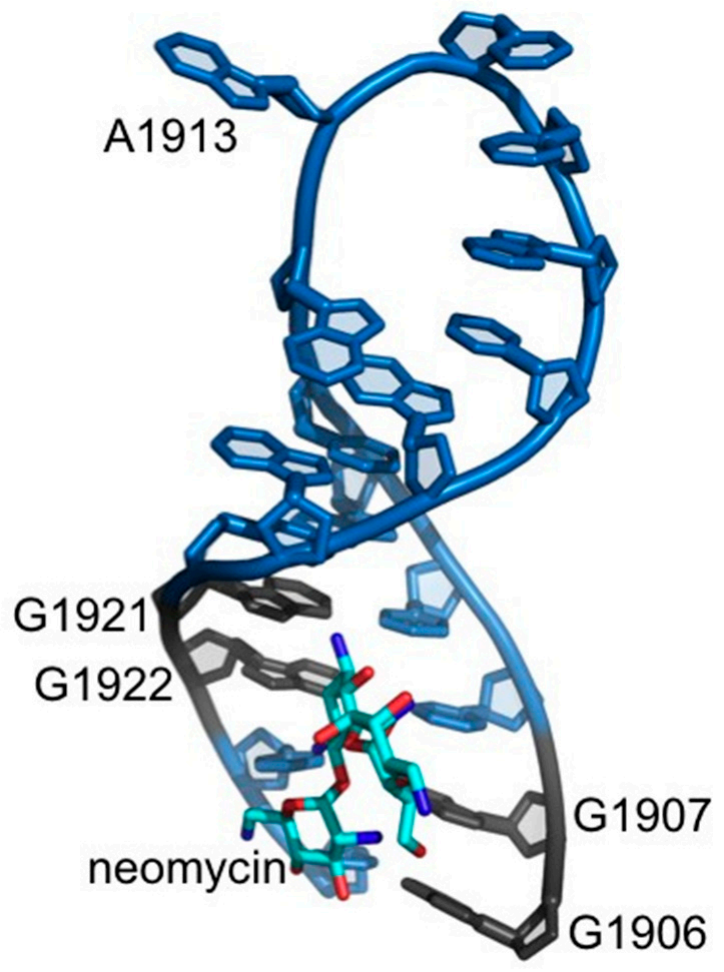
The interactions of H69 with neomycin are shown. Neomycin (cyan) interacts through H69 residues G1906, G1921, and G1922. Residue A1913 is flipped out from the loop. The residues that coordinate to cisplatin are shown in grey and the remaining residues are in blue. The figure was created by using PDB file 4GAQ [50].

The observation that residues in H69 have altered DMS or RNase sensitivity upon platination suggests that structural alterations in the hairpin loop region have occurred. This effect on the loop residues upon cisplatin coordination at distant stem GpG sites is not surprising since platination of DNA causes significant distortion of the backbone [48]. In addition, other drug molecules have been shown to bind to the H69 stem and cause structural changes in the loop region. Aminoglycoside binding to a 2-aminopurine-modified H69 was shown to affect the loop nucleotides, in which residue A1913 became more solvent exposed [49]. These results were consistent with X-ray crystal structures of bacterial 70S ribosomes with neomycin bound to the stem region of H69 near residues G1906, 1921, and 1922 and base exposure of A1913 (Figure 7) [50]. Therefore, complex **1** coordination to the same residues, as revealed in the present study, could induce structural changes in H69 in both the stem and loop regions. In the 2-aminopurine modified H69 studies, structural changes in the loop region were prominent only for the modified H69 (Ψ containing), whereas in the current work, platination of both unmodified and modified H69 RNAs caused similar effects on the loop (*i.e*., altered DMS or RNase sensitivity). These results support the expected conclusion that impacts on RNA conformation would depend on the drug types and their binding modes, particularly with respect to specific nucleotide contacts.

Many structural effects such as bending, unwinding, helix destabilization, and base destacking have been shown to result from cisplatin binding to DNA [51]. Likewise, the current study showed structural changes due to cisplatin coordination to the rRNA hairpins. The structural effects of platination on DNA have been suggested to interfere with replication, transcription, and repair. Similarly, the structural effects of platination on rRNA could interfere with protein synthesis and impact drug cytotoxicity. The modified and unmodified H69 RNAs gave similar results for reactions with complex **1** in which stem GpG sites were targeted. The two target GpG sites in H69 are involved in either G-C or G•U pairs within a duplex region. Similar reactivity was observed with the 790 loop in which a stem GpG site was targeted, although only one adduct was identified in this case. Unmodified H69 and the 790 loop have identical types and numbers of nucleotides (A_5_G_5_U_4_C_5_), but different sequences, with the 790 loop containing one and H69 having two GpG sites. Chemical probing of both H69 RNAs showed platination-induced DMS sensitivity in the loop region, whereas no such effect was observed for the 790 loop. Furthermore, probing of modified and unmodified H69 revealed that these two constructs have similar reactivities with complex **1** and DMS, suggesting similar solvent accessibilities and chemical properties. Therefore, unlike the case of aminoglycosides, a minimal influence from pseudouridines on structural changes induced by cisplatin coordination was seen. Collectively, these data indicate a greater impact of the primary nucleotide sequences than the modified nucleotides on platination and subsequent secondary structural rearrangements.

Structural flexibility of H69 and h24 is important during protein synthesis as both motifs are involved in forming intersubunit bridges [25]. H69 compresses by ~5 Å to maintain the intersubunit contact during the ratchet-like movement in protein synthesis, and stabilization of the compressed form by neomycin binding is suggested to interfere with translocation [37,52]. Similarly, bridge B2b formed between h24 and H67/H69 likely needs to be maintained during various stages of translation. Drug binding to key residues involved in these intersubunit contacts could alter the conformational states of these dynamic motifs and inhibit protein synthesis [38]. Therefore, both sequence and pseudouridine-dependent drug-induced (e.g., aminoglycosides or cisplatin) structural changes in rRNA are likely to impact the translation process.

The initial goal of this work was to compare two rRNA hairpins with the same nucleotide content (*i.e*., A_5_G_5_U_4_C_5_) and similar locations at the subunit interface. It should be noted, however, that prior work to identify platination sites by complex **1** on *E. coli* 16S rRNA (in the context of free RNA, 30S subunits, and 70S ribosomes) through primer extension analysis also suggested adduct formation at the G786 site within the 790 loop. In contrast, the primer extension method could not confirm the bis-adduct (e.g., GpG) as revealed in the current work through MALDI MS and DMS probing [14]. Furthermore, in the earlier work on 16S rRNA, G786 was a minor site compared to stronger reactivity by complex **1** at G799/G800, which reside at a helix-loop junction. The minor reactivity at G786 was also not observed in the context of complete 70S ribosomes. The neighboring G800 residue was not present in the model 790 hairpin used in this work, suggesting that cisplatin may prefer GpG sites in more accessible secondary structure motifs compared to the stem regions [14,53]. These comparisons also indicate that complex **1** interactions with rRNA are dependent on the context (e.g., individual subunits *vs.* complete ribosomes), thus supporting the hypothesis that the structure of these sites varies with subunit association. Similarly, platination of rRNA at functionally important sites could cause distortions of secondary structure motifs and/or impact RNA structural dynamics, therefore interfering with subunit association, ribosome function, and drug cytotoxicity. Interestingly, recent studies by Hostetter and coworkers showed higher platination events on cellular RNA (also with an emphasis on rRNA) compared to DNA in yeast [16]. Similar investigations with mammalian cells combined with “click”-modified analog reactions to isolate the platinated species [54] and high-throughput sequencing approaches [55] will allow determination of the locations of platinum adducts on a much broader range of RNAs (e.g., mRNA, non-coding RNAs, *etc.*) as well as a better understanding of their biological impacts.

## 3. Experimental Section

### 3.1. Buffers

Standard aqueous buffer solutions were prepared from KH_2_PO_4_ and K_2_HPO_4_ (Fisher Scientific, Waltham, MA, USA), NaClO_4_ (Sigma, Saint Louis, MO, USA), and Millipore water (ddH_2_O). The following buffers were employed: buffer A (10 mM KH_2_PO_4_/K_2_HPO_4_, pH 6.2, 20 mM NaClO_4_), buffer B (10 mM KH_2_PO_4_/K_2_HPO_4_, pH 6.2, 20 mM NaCl), 10× TBE (89 mM Tris-HCl, 89 mM boric acid, 2.5 mM EDTA, pH 8.3), and denaturing loading buffer (0.1% bromophenol blue, 0.1% xylene cyanol, 1× TBE, 8 M urea).

### 3.2. Metal Complexes

Cisplatin, *cis*-[PtCl_2_(NH_3_)_2_], was obtained from Alfa Aesar (Ward Hill, MA, USA). Silver nitrate was purchased from Fisher Scientific. Dimethylformamide (DMF) was obtained from Acros Organics. The activated complex *cis*-[PtCl(NH_3_)_2_X]^+/^°, in which X is DMF or NO_3_^−^, was prepared by mixing 1:1 equivalents of cisplatin and AgNO_3_ dissolved in DMF and agitating in the dark overnight at 37 °C [56]. The resulting AgCl precipitate was removed by repeated centrifugation. For all studies, the activated (monoaquated) complex **1** was freshly prepared by dilution of *cis*-[PtCl(NH_3_)_2_X]^+/^° to intermediate stock concentrations with water just prior to the experiments.

### 3.3. Nucleic Acids

All RNA constructs were obtained from Thermo Fisher Scientific. Concentrations of RNA solutions were calculated using the absorbance at 260 nm and single-stranded extinction coefficients obtained by the nearest-neighbor approach (H69 modified or unmodified ε_260 nm_ = 189,400 M^−1^·cm^−1^; 790 loop ε_260 nm_ = 188,800 M^−1^·cm^−1^) [24,30].

### 3.4. End Labeling of Ribonucleic Acid (RNA) Constructs

RNA constructs were radiolabeled at the 3′ end with T4 RNA ligase (New England Biolabs, Ipswich, MA, USA) and [5′-^32^P]-pCp (Perkin-Elmer Life Sciences, Inc., Waltham, MA, USA) [57]. The labeling was performed in T4 RNA ligase buffer (50 mM Tris-HCl, pH 7.5, 10 mM MgCl_2,_ 1 mM dithiothreitol) with 50 pmol RNA, 10 μCi of [5′-^32^P]-pCp, 12 units of T4 RNA ligase, and 1 mM ATP in a 30 μL reaction at 4 °C overnight. Labeled RNAs were ethanol precipitated and purified on 20% denaturing (8 M urea) polyacrylamide gels. The labeled products were visualized by autoradiography, then excised and eluted by the “crush and soak” method in 350 mM NaOAc, pH 5.3, 0.1 mM EDTA buffer overnight at 4 °C. The extracted RNAs were desalted over C18 Sep-Pak cartridges (Waters, Milford, MA, USA).

RNA constructs were 5′-end labeled in T4 polynucleotide kinase buffer (70 mM Tris-HCl, pH 7.6, 10 mM MgCl_2_, 5 mM dithiothreitol) (New England Biolabs) with 10 μCi of [γ-^32^P]-ATP (Perkin-Elmer Life Sciences, Inc., Waltham, MA, USA) and 3 units of T4 polynucleotide kinase (New England Biolabs) in a total volume of 30 μL [58]. After incubation at 37 °C for 30 min, the RNAs were ethanol precipitated and purified as described for 3′-end labeling.

### 3.5. Large-Scale Platination and RNase T1 Mapping

The platination reactions of all RNA constructs for RNase T1 mapping studies were performed in buffer B. Prior to platination, the RNAs were renatured by placing the sample tubes in a boiling water bath for 2 min followed by placement on ice. Platination was carried out in the dark at 37 °C for 5 h with a 1:2 ratio of RNA:complex **1** and 3 nmol RNA (90 μM complex **1**). The RNA products were separated on 20% denaturing polyacrylamide gels. The RNA bands were visualized by UV shadowing and excised from the gel. Bands with slower mobility than the unmodified RNAs were assigned as the platinated products. The platinated RNA products were eluted in 550 mM NH_4_OAc, pH 5.5, 0.1 mM EDTA buffer and desalted in C18 Sep-Pak cartridges. RNase T1 digestion of platinated and unplatinated RNAs was performed in water for 20 min at 37 °C using 1 unit of enzyme (Sigma). Digested samples were dried and resuspended in deionized water, mixed with saturated 3-hydroxy picolinic acid in 50% acetonitrile, and spotted on a MALDI plate. MALDI-MS analysis was performed on a Bruker Daltonics TOF-300 MALDI Ultraflex instrument equipped with a nitrogen laser (λ 337 nm). The mass spectra of all RNAs were acquired in the positive-ion reflector mode. Peak masses were assigned using Flex Analysis version 2.0 software (Bruker Daltonics, Billerica, MA, USA).

For chemical probing, gel-purified, radiolabeled RNAs (8 × 10^5^–10 × 10^5^ cpm) and unlabeled RNAs (0.7 μM) were combined and renatured in buffer A. Platination reactions were initiated by adding complex **1** (final concentration 94 μM) to RNA in a final reaction volume of 60 μL. The reactions were incubated in the dark at 37 °C for 3 h. Control RNA samples did not contain platinum complex. Platinated RNA products and unreactive RNAs were separated on 20% polyacrylamide gels, followed by elution with the “crush and soak” method.

### 3.6. Chemical Probing of Platinated RNAs

Guanosine-specific reactions [45] on the platinated RNAs were performed in dimethyl sulfate (DMS) buffer (50 mM sodium cacodylate, pH 5, 1 mM EDTA) in the presence of 5 μg of carrier tRNA. The reactions were initiated by addition of 1 μL of 50% (*v*/*v*) DMS in water and incubating for 60 s at 90 °C, followed by immediate quenching with stop solution (1.5 mM sodium acetate, pH 7, 1 M β-mercaptoethanol). The RNAs were ethanol precipitated, redissolved in 10 μL of 1 M Tris-HCl, pH 8.2, and then treated with 10 μL of freshly prepared 0.2 M NaBH_4_ and incubated on ice for 30 min in the dark. The RNAs were ethanol precipitated again and treated with 10 μL of 1 M aniline acetate, pH 4.5, at 60 °C for 30 min in the dark. The samples were dried, lyophilized twice in water, and resuspended in denaturing loading buffer. Samples (approximately 2 × 10^4^ cpm) were run on high-resolution sequencing gels (20% denaturing polyacrylamide gels, 0.4 mm thickness, 1× TBE, 8 M urea) and visualized by autoradiography.

Alkaline hydrolysis of RNA constructs labeled at the 3′ or 5′ ends (1 × 10^4^ cpm) was done by mixing with buffer (30 mM NaOH, 0.3 mM EDTA) and boiling for 90 s followed by quick freezing on dry ice. The samples were thawed and mixed with denaturing loading buffer just prior to electrophoresis.

RNase T1 digestion was carried out on end-labeled RNA (1 × 10^4^ cpm) by mixing with buffer (20 mM sodium citrate, pH 5.0, 7 mM urea, 1 mM EDTA) and 0.5 units of RNase T1 followed by incubation at 55 °C for 20 min. The reaction products were mixed with denaturing loading buffer and loaded directly onto 20% denaturing gels.

## 4. Conclusions

The rRNA motifs investigated in this study are highly conserved throughout phylogeny and important for proper ribosome function [25,26,33]. Therefore, similar platination events on eukaryotic ribosomes could play a role in drug toxicity. The loop Ψs in H69 regulate RNA structure, dynamics, and stability [23,30,31,32,49]. In this work, platination of modified H69, unmodified H69, and the 790 loop was probed by using RNase T1 mapping and DMS reactions. Activated cisplatin, or complex **1**, reacted with the three 19-nucleotide rRNA motifs in a similar manner. Platination occurred at consecutive Gs, namely G1921-G1922 and G1906-G1907 in the stem region of H69 (unmodified and pseudouridylated), and at G785-G786 of the 790 loop, most likely as bis-adducts, in which the chlorido ligands are displaced by two G residues. Previous studies with truncated tRNA molecules showed a preference for platination at neighboring Gs within a G-C-rich wobble-base-pair region [13]. Therefore, consistent with prior RNA studies, this work shows preferential recognition of G residues in rRNA motifs by complex **1**. Due to the relative simplicity of the reactive motif (GpG), similar platination sites may occur on other RNAs such as mRNA, tRNA, and non-coding RNAs *in vivo*.

Chemical probing studies also revealed structural alterations in the RNA constructs following drug coordination, as well as the influence of nucleotide sequence on such changes. Comparisons to previous reports on rRNA-cisplatin interactions indicate that the GpG locations within the complete folded secondary structure will impact the reactivity, and not surprisingly, the presence of proteins and RNA-RNA contacts will also play a role. Overall, the data provided here show many similarities of cisplatin coordination to DNA and RNA, such as target preference (e.g., GpG) and impact on structure. Further studies may reveal how these adducts could further impact the biological function of rRNAs.
